# Qingyi decoction combined with western medicine comprehensive therapy for acute pancreatitis: intestinal barrier protection and sepsis prevention—a retrospective clinical study

**DOI:** 10.3389/fcell.2026.1892868

**Published:** 2026-09-07

**Authors:** Jia Ai, Xudong Yin, Zhi Yao

**Affiliations:** 1 Department of Traditional Chinese Medicine, People’s Hospital of Tongliang District, Chongqing, China; 2 Department of Hepatobiliary and Pancreatic Surgery, People’s Hospital of Tongliang District, Chongqing, China

**Keywords:** acute pancreatitis (AP), bacterial translocation, intestinal barrier function, Qingyi decoction, sepsis prevention, sepsis, traditional Chinese medicine

## Abstract

**Background:**

Acute pancreatitis (AP) is a common acute abdominal condition with high morbidity, mortality, and treatment costs. Approximately 20% of patients progress to moderately severe or severe acute pancreatitis (MSAP/SAP), with mortality rates reaching 20%–40%. Sepsis, as a critical and life-threatening complication of severe acute pancreatitis, significantly worsens prognosis through intestinal barrier dysfunction and bacterial translocation. The pathophysiology of AP involves premature activation of pancreatic enzymes leading to acinar cell death, including apoptosis, necrosis, and pyroptosis, which trigger the inflammatory cascade and subsequent systemic complications. Traditional Chinese medicine (TCM), particularly Qingyi Decoction combined with Western medicine comprehensive therapy, has shown promising effects in alleviating abdominal symptoms, activating intestinal physiological function, and reducing the severity rate of AP. However, clinical studies specifically evaluating sepsis prevention and modulation of cell death pathways through intestinal barrier protection are limited.

**Aim:**

To retrospectively evaluate the clinical efficacy and safety of Qingyi Decoction (Qingyi Tang combined with Dachaihu Tang) combined with Western medicine comprehensive therapy in the treatment of mild to moderate acute pancreatitis, with a primary focus on its protective effects against sepsis progression through intestinal barrier function improvement and modulation of cell death pathways.

**Methods:**

A single-center, retrospective clinical study was conducted from January 2024 to December 2025 (data lock date: 31 December 2025) at Tongliang District People’s Hospital. A total of 108 patients with mild to moderate acute pancreatitis (intestinal damp-heat syndrome) were enrolled and divided into two groups: the Group A (Qingyi Decoction enema and oral administration combined with Western medicine comprehensive therapy, n = 54) and the Group B (Western medicine comprehensive therapy alone, n = 54). Primary outcomes included TCM syndrome scores, modified CT severity index (MCTSI) scores, and intestinal barrier function indicators (D-lactate, diamine oxidase, endotoxin). Secondary outcomes encompassed inflammatory markers (CRP, IL-6, white blood cells, neutrophils), SIRS scores, antibiotic usage rate and duration, length of hospital stay, hospitalization costs, and sepsis incidence defined by Sepsis-3 criteria. Assessments were performed at baseline, day 3 of hospitalization, and before discharge.

**Results:**

The Group A demonstrated significantly greater improvements in TCM syndrome scores compared to Group B at day 3 (MD = −4.82, 95%CI: −6.15 to −3.49, p < 0.001) and before discharge (MD = −6.35, 95%CI: −7.89 to −4.81, p < 0.001). MCTSI scores were significantly lower in Group A at day 3 (MD = −1.25, 95%CI: −1.78 to −0.72, p < 0.001) and before discharge (MD = −1.68, 95%CI: −2.31 to −1.05, p < 0.001). Intestinal barrier function markers including D-lactate (MD = −2.15, 95%CI: −2.89 to −1.41, p < 0.001), diamine oxidase (MD = −3.42, 95%CI: −4.28 to −2.56, p < 0.001), and endotoxin (MD = −0.18, 95%CI: −0.25 to −0.11, p < 0.001) were significantly improved in the treatment group. Inflammatory markers CRP (MD = −28.5, 95%CI: −36.2 to −20.8, p < 0.001) and IL-6 (MD = −15.3, 95%CI: −19.7 to −10.9, p < 0.001) showed significant reductions. Most notably, the sepsis incidence was significantly lower in Group A (3.7% vs. 14.8%, OR = 0.22, 95%CI: 0.05–0.98, p = 0.046), representing a 75% relative risk reduction. Antibiotic usage rate (44.4% vs. 66.7%, p = 0.021), antibiotic duration (MD = −3.5 days, 95%CI: −4.8 to −2.2, p < 0.001), length of hospital stay (MD = −4.2 days, 95%CI: −5.6 to −2.8, p < 0.001), and hospitalization costs (MD = −4,850 yuan [approximately USD 683, based on average RMB/USD exchange rate of 7.1 for 2024–2025], 95%CI: −6,320 to −3,380, p < 0.001) were all significantly reduced in the treatment group.

**Conclusion:**

Qingyi Decoction combined with Western medicine comprehensive therapy significantly improves clinical symptoms, reduces inflammatory response, enhances intestinal barrier function, markedly decreases sepsis incidence from 14.8% to 3.7% (75% relative risk reduction), and shortens hospitalization duration and costs in patients with mild to moderate acute pancreatitis. This integrated approach represents a promising therapeutic strategy for AP management and sepsis prevention through intestinal barrier protection.

## Introduction

Acute pancreatitis (AP) is one of the most common acute abdominal conditions encountered in clinical practice (Banks et al., 2013). According to the Chinese Guidelines for the Diagnosis and Treatment of Acute Pancreatitis (2019) (Pancreatic Diseases Group et al., 2019), AP is classified into mild acute pancreatitis (MAP), moderately severe acute pancreatitis (MSAP), and severe acute pancreatitis (SAP). While most patients present with mild, self-limiting disease that typically resolves within 1 week, approximately 20% progress to moderate or severe forms characterized by pancreatic or peripancreatic tissue necrosis, organ failure, or both, with mortality rates ranging from 20% to 40% (Schepers et al., 2019; Gurusamy et al., 2016).

The pathophysiology of severe acute pancreatitis involves a complex cascade of inflammatory responses initiated by premature activation of pancreatic enzymes within acinar cells, leading to autodigestion of the pancreas and surrounding tissues. This process triggers multiple cell death pathways, including apoptosis, necrosis, and pyroptosis, which play pivotal roles in disease progression and severity. When AP triggers systemic complications, patients may develop systemic inflammatory response syndrome (SIRS), sepsis, and multiple organ dysfunction syndrome (MODS) (Petrov et al., 2010). Sepsis, in particular, represents a critical and often fatal complication that significantly worsens prognosis. The presence of extensive peripancreatic effusion, intestinal paralysis, intra-abdominal hypertension (IAH), and abdominal compartment syndrome (ACS) substantially increases mortality rates, and currently, no optimal preventive treatment strategy exists for these severe manifestations (Cao and Tang, 2014; Zeng and Du, 2020).

The gut has been increasingly recognized as a key player in the pathogenesis of severe acute pancreatitis and sepsis. Intestinal barrier dysfunction allows bacterial translocation from the gut lumen to systemic circulation, triggering and perpetuating the inflammatory cascade that leads to sepsis (Li et al., 2020). D-lactate, diamine oxidase (DAO), and endotoxin serve as critical biomarkers reflecting intestinal permeability, mucosal integrity, and bacterial translocation, respectively. Notably, intestinal epithelial cell death, including apoptosis and pyroptosis, contributes to barrier disruption and bacterial translocation in severe AP. Elevated levels of these markers are strongly associated with increased sepsis risk and poor prognosis in AP patients. Early intervention to restore gastrointestinal function, maintain intestinal barrier integrity, and modulate cell death pathways has therefore emerged as a crucial therapeutic strategy for sepsis prevention. Studies have demonstrated that early gastrointestinal functional therapy can inhibit bacterial overgrowth, protect intestinal function, alleviate clinical symptoms, and reduce complication rates (Zhu et al., 2017). Enteral nutrition, when tolerated, has been shown to be safe and effective in reducing infectious complications, MODS incidence, and mortality compared to parenteral nutrition (Wang and Lu, 2016).

Traditional Chinese medicine (TCM) has a long history in treating abdominal disorders and has shown promising results in AP management. According to TCM theory, acute pancreatitis belongs to the categories of “abdominal pain,” “vomiting,” “pancreas fever,” and “stomach heart pain.” The disease is characterized by qi stagnation, damp-heat, blood stasis, food stagnation, and stone accumulation in the middle jiao, leading to dysfunction of the spleen and stomach, generation of damp-heat, and clinical manifestations of abdominal distension, pain, and vomiting (Chinese Pancreatic Surgery Association et al., 2021). The disease location is in the pancreas, closely related to the liver, gallbladder, stomach, and intestines, and involves the lungs, heart, kidneys, and brain.

The treatment principle follows the concept of “treating blockage with passage” (tongze buyong), as the pathogenesis is characterized by interior, heat, and excess patterns. Various TCM formulas have demonstrated efficacy in AP treatment. Dachengqi Tang has been shown to alleviate pancreatic injury and promote intestinal mucosal barrier repair through anti-inflammatory mechanisms (Tang et al., 2021). Recent studies suggest that TCM formulas may also modulate cell death pathways, including inhibition of excessive apoptosis and pyroptosis in pancreatic acinar and intestinal epithelial cells. Chaisha Chengqi Tang combined with somatostatin has demonstrated significant efficacy in reducing serum inflammatory cytokine levels and improving clinical symptoms (Xu, 2016). Chaikin Chengqi Tang has also shown favorable therapeutic effects in AP management (Zhang, 2018).

Qingyi Decoction, a modified combination of Qingyi Tang and Dachaihu Tang, has been employed in multiple clinical studies for the treatment of acute pancreatitis with intestinal damp-heat syndrome (Wang G. et al., 2022; Wang GY. et al., 2022; Chen et al., 2015). The formula has demonstrated clinical efficacy in improving gastrointestinal function, reducing inflammatory burden, and alleviating abdominal symptoms in AP patients across various Chinese institutions. The formula comprises Honghua (Carthamus tinctorius), Taoren (Prunus persica), Chuanxiong (Ligusticum chuanxiong), Shanzha (Crataegus pinnatifida), Chaobaizhu (Atractylodes macrocephala), Yanhusuo (Corydalis yanhusuo), Chuanlianzi (Melia toosendan), Shengshigao (Gypsum fibrosum), Dahuang (Rheum palmatum), Mangxiao (Natrii sulfas), Yinhua (*Lonicera japonica*), Peilan (Eupatorium fortunei), Zhishi (Citrus aurantium), Houpo (Magnolia officinalis), Baishao (Paeonia lactiflora), Huangqin (Scutellaria baicalensis), and Chaihu (Bupleurum chinense). The formula functions to purge the bowels, clear heat, and promote qi movement, with heavy dosage of Dahuang (30 g) to achieve the desired therapeutic effect. Modern pharmacological studies have demonstrated that several components of Qingyi Decoction, including emodin from Dahuang and baicalin from Huangqin, possess regulatory effects on cell death pathways, including inhibition of NLRP3 inflammasome-mediated pyroptosis and modulation of mitochondrial apoptosis.

Previous preliminary studies with 32 AP patients demonstrated that Qingyi Decoction enema and oral administration could significantly alleviate abdominal distension and pain symptoms, accelerate C-reactive protein (CRP) improvement, shorten antibiotic treatment duration, reduce hospitalization time, decrease complications, and improve quality of life (Zhang et al., 2015). However, these findings were limited by small sample size and lack of rigorous randomized controlled design.

This retrospective clinical study was designed to evaluate the efficacy and safety of Qingyi Decoction combined with Western medicine comprehensive therapy in the treatment of mild to moderate acute pancreatitis, with a primary focus on sepsis prevention through intestinal barrier function improvement and modulation of cell death pathways. We hypothesized that Qingyi Decoction would protect intestinal barrier integrity, reduce bacterial translocation, attenuate systemic inflammation, modulate excessive cell death, and thereby significantly lower sepsis incidence compared to Western medicine alone.

## Materials and methods

### Study design and participants

This was a single-center, retrospective clinical study conducted at Tongliang District People’s Hospital, Chongqing, China, from January 2024 to December 2025 (data lock date: 31 December 2025). The study protocol was approved by the Ethics Committee of Tongliang District People’s Hospital (Approval No. 2023-LunShen-No.44). All participants provided written informed consent before enrollment.

A total of 108 patients with mild to moderate acute pancreatitis were enrolled and divided into two groups based on treatment received: Group A (n = 54) and Group B (n = 54). Patients in Group A received Qingyi Decoction combined with Western medicine comprehensive therapy, while those in Group B received Western medicine comprehensive therapy alone. The sample size was calculated based on the sepsis incidence observed in prior studies: an expected sepsis rate of 15% in the Qingyi Decoction group versus 35% in the control group, using a two-sided alpha of 0.05 and a statistical power of 80%. Using the formula for two-proportion comparison (n = [Zα/2 + Zβ]^2^ × [p1 (1-p1) + p2 (1-p2)]/(p1-p2)^2^), a minimum of 47 patients per group was required. The calculated minimum sample size was 47 patients per group, and 54 eligible patients per group were ultimately included, yielding a total of 108 patients. This calculation method has been previously employed in comparable TCM-Western medicine combined intervention trials for acute pancreatitis (Ji et al., 2016). Supplementary power analyses were conducted for the co-primary endpoints: for TCM syndrome scores, assuming a clinically meaningful between-group difference of 4 points at day 3 (SD ≈ 3.1, two-sided α = 0.05, power = 80%), approximately 20 patients per group were required, indicating that the enrolled sample of 54 per group is substantially overpowered for this endpoint. For MCTSI scores, assuming a between-group difference of 1.0 point (SD ≈ 1.3, same parameters), approximately 28 patients per group were required, again well within the capacity of the enrolled sample. The study was thus primarily powered for the sepsis endpoint as the most clinically critical and statistically demanding outcome, while remaining adequately powered for the continuous co-primary endpoints.

### Inclusion criteria

1. Age 18–75 years, male or female.
2. Diagnosis of acute pancreatitis according to the Chinese Guidelines for the Diagnosis and Treatment of Acute Pancreatitis (2021) (Li et al., 2021; Chinese Pancreatic Surgery Association et al., 2021): (a) persistent upper abdominal pain; (b) serum amylase and/or lipase levels at least 3 times above the upper limit of normal; (c) characteristic findings of acute pancreatitis on abdominal imaging. At least 2 of 3 criteria must be met.
3. Classification as mild to moderate AP according to the revised Atlanta classification (RAC) and modified CT severity index (MCTSI) scoring system.
4. TCM syndrome differentiation as intestinal damp-heat pattern according to the Guidelines for Clinical Research of New Chinese Medicines (Zheng, 2002).
5. Patients with common etiologies (biliary stone disease, hyperlipidemia, alcohol, or diabetes) were NOT excluded *per se*; however, those whose pancreatitis was considered to be primarily etiology-driven with pre-existing uncontrolled comorbidities (e.g., uncontrolled hyperlipidemia >11.3 mmol/L, cholangitis requiring urgent ERCP) were excluded if such conditions would significantly confound the assessment of TCM treatment efficacy or necessitate additional therapeutic interventions beyond the study protocol.
6. Provision of written informed consent.

#### Exclusion criteria

1. Severe acute pancreatitis (SAP).
2. Severe cardiovascular or cerebrovascular diseases.
3. Malignant tumors.
4. Allergy to study medications.
5. Recent use of antibiotics or immunosuppressive drugs.
6. Pregnancy or lactation.
7. Participation in other clinical trials within 3 months. Clinical deterioration or inability to tolerate any form of oral or enema administration after the initial 3-day enema phase (e.g., worsening MCTSI score, progression to SAP, or hemodynamic instability requiring ICU-level care).

## Treatment protocol

Patients were divided into two groups based on the treatment protocol they received. Group A received standard Western medicine comprehensive therapy plus Qingyi Decoction enema and oral administration. Group B received standard Western medicine comprehensive therapy alone.

### Group A (Qingyi decoction + western medicine)

Patients in Group A received standard Western medicine comprehensive therapy plus Qingyi Decoction enema and oral administration. The Qingyi Decoction formula consisted of: Honghua 15g, Taoren 15g, Chuanxiong 15g, Shanzha 30g, Chaobaizhu 15g, Yanhusuo 15g, Chuanlianzi 15g, Shengshigao 30g, Dahuang 30g, Mangxiao 9 g (dissolved into the prepared decoction immediately before administration), Yinhua 15g, Peilan 15g, Zhishi 15g, Houpo 15g, Baishao 15g, Huangqin 15g, and Chaihu 15 g.

Preparation: The above herbs were decocted with 1,500 mL water for 45 min using an automatic decoction machine, filtered to remove residue, and concentrated to 300 mL.

Administration: Enema was performed twice daily (morning and evening) starting from the first day of hospitalization, with the decoction retained for 15–20 min. Enema administration was continued for 3 days (Days 1–3) as the initial phase. On Day 4, clinical re-evaluation was performed: patients who demonstrated clinical improvement (reduction of at least 30% in abdominal pain VAS score, tolerance to oral intake, and absence of clinical deterioration) were transitioned to combined enema plus oral administration of the decoction (divided into morning and evening doses before meals on an empty stomach) until day 5–7 or discharge. Patients who did not meet these criteria for oral tolerance by Day 4 continued enema-only administration and were flagged for clinical re-assessment. Patients whose condition deteriorated (as defined in the Exclusion Criteria) were withdrawn from the study protocol and managed per clinical judgment. Western medicine treatment remained unchanged throughout.

### Group B (Western medicine alone)

Patients received standard Western medicine comprehensive therapy according to the Chinese Guidelines for the Diagnosis and Treatment of Acute Pancreatitis (2021), including fluid resuscitation, pain management, nutritional support, and organ function monitoring. Antibiotic use was determined according to clinical indications, defined per the 2021 Chinese Guidelines for the Diagnosis and Treatment of Acute Pancreatitis: antibiotic therapy was initiated only in the presence of documented or clinically suspected infection, operationally defined as fever persisting beyond 72 h combined with imaging evidence of pancreatic or peripancreatic infection, positive blood cultures, or clinical deterioration suggesting secondary infection. Prophylactic antibiotic use in the absence of these criteria was not performed.

## Outcome measures

### Primary outcomes

1. Traditional Chinese Medicine (TCM) syndrome score (Table 1): Assessed using a validated TCM syndrome scoring scale for intestinal damp-heat pattern in acute pancreatitis, including major symptoms (abdominal pain, fever and thirst, chest tightness and abdominal distension) and minor symptoms (headache and body heaviness, nausea and vomiting, loose stools), scored as none (0 points), mild (2 points), moderate (4 points), and severe (6 points).
2. Modified CT Severity Index (MCTSI) score (Table 2): Evaluated by abdominal CT scan at baseline, day 3, and before discharge.
3. Intestinal barrier function markers: Plasma D-lactate, diamine oxidase (DAO), and endotoxin levels measured by enzymatic spectrophotometry at baseline, day 3, and before discharge.

**TABLE 1:** Comparison of Traditional Chinese Medicine syndrome scores between groups.

Time point	Group A (n = 54)	Group B (n = 54)	Mean difference (95%CI)	*p-value*
Baseline	18.6 ± 3.4	18.2 ± 3.7	0.4 (−0.9–1.7)	0.558
Day 3	8.5 ± 2.8	13.3 ± 3.1	−4.82 (−6.15 to −3.49)	<0.001
Before discharge	3.2 ± 1.6	9.5 ± 2.4	−6.35 (−7.89 to −4.81)	<0.001

Data are presented as mean ± standard deviation. CI: confidence interval.

**TABLE 2:** Comparison of modified CT severity index scores between groups.

Time point	Group A (n = 54)	Group B (n = 54)	Mean difference (95%CI)	*p-value*
Baseline	4.2 ± 1.3	4.1 ± 1.4	0.1 (−0.4–0.6)	0.702
Day 3	2.8 ± 1.1	4.1 ± 1.2	−1.25 (−1.78 to −0.72)	<0.001
Before discharge	1.5 ± 0.8	3.2 ± 1.0	−1.68 (−2.31 to −1.05)	<0.001

Data are presented as mean ± standard deviation. CI: confidence interval.

### Secondary outcomes

1. Systemic Inflammatory Response Syndrome (SIRS) score (Table 3): Assessed at baseline and 48 h for SAP prediction.
2. Inflammatory markers: C-reactive protein (CRP), interleukin-6 (IL-6), white blood cell count, neutrophil count, neutrophil percentage, serum creatinine, blood urea nitrogen, and serum amylase (AMS).
3. Sepsis incidence: Defined according to the Sepsis-3 criteria (life-threatening organ dysfunction caused by a dysregulated host response to infection) (Singer et al., 2016). SIRS was defined using the standard four-criterion definition: (1) body temperature >38 °C or <36 °C; (2) heart rate >90 beats per minute; (3) respiratory rate >20 breaths per minute or PaCO_2_ <32 mmHg; and (4) white blood cell count >12 × 10^9^/L or <4 × 10^9^/L, or >10% immature band forms. SIRS was diagnosed when two or more criteria were present.
4. Antibiotic usage rate and duration.
5. Length of hospital stay.
6. Hospitalization costs.

**TABLE 3:** Comparison of systemic inflammatory response syndrome scores between groups.

Time point	Group A (n = 54)	Group B (n = 54)	*p-value*
Baseline SIRS score, mean ± SD	3.2 ± 0.8	3.1 ± 0.9	0.548
SIRS positive at baseline, n (%)	48 (88.9)	46 (85.2)	0.563
Persistent SIRS at 48 h, n (%)	9 (16.7)	20 (37.0)	0.018
SIRS resolved before discharge, n (%)	52 (96.3)	43 (79.6)	0.008

SD: standard deviation; SIRS: systemic inflammatory response syndrome.

## Statistical analysis

Statistical analysis was performed using R software (version 4.3.0, R Foundation for Statistical Computing, Vienna, Austria) and SPSS 26.0 (IBM Corp., Armonk, NY, United States of America). R packages including ‘tableone’, ‘ggplot2’, and ‘rms’ were used for data processing, visualization, and regression modeling. Continuous variables were expressed as mean ± standard deviation (SD) and compared between groups using independent t-tests or Mann-Whitney U tests depending on data distribution. Categorical variables were expressed as frequencies (percentages) and compared using chi-square tests or Fisher’s exact tests. Repeated measures analysis of variance (ANOVA) was used to compare outcomes at different time points. A two-sided p-value <0.05 was considered statistically significant.

## Results

### Study flow and baseline characteristics

**FIGURE 1:**
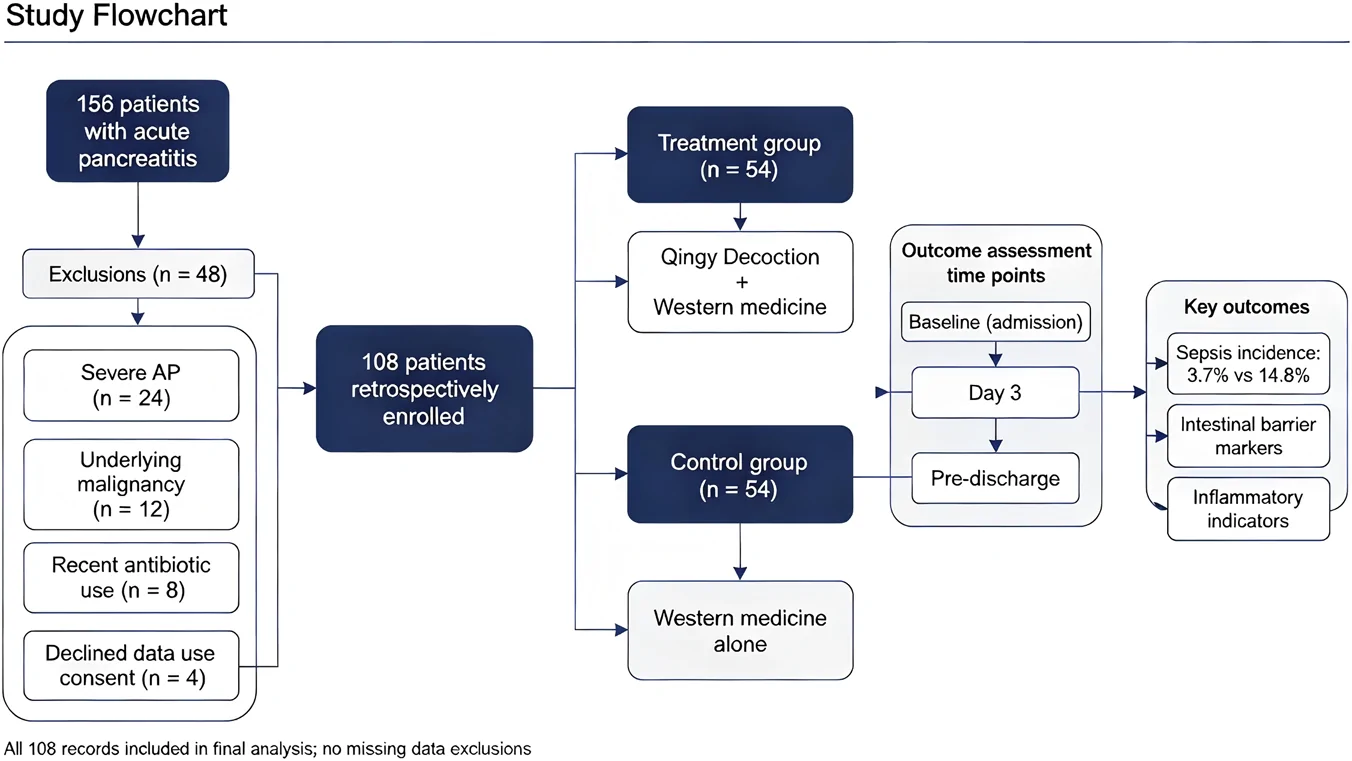
Flow diagram showing 156 patients with acute pancreatitis screened, with 48 excluded because of severe acute pancreatitis, underlying malignancy, recent antibiotic use, or refusal to participate. The remaining 108 patients were allocated according to treatment received into Group A, receiving Qingyi Decoction plus Western medicine, and Group B, receiving Western medicine alone, with 54 patients in each group. All included patients had complete outcome data. Assessments were performed at baseline, day three, and before discharge.

A total of 156 patients with acute pancreatitis were screened for eligibility from January 2024 to December 2025. Of these, 48 patients were excluded (24 with severe AP, 12 with underlying malignancy, 8 with recent antibiotic use, and 4 who declined participation). The remaining 108 patients were allocated by treatment received into Group A (n = 54) and Group B (n = 54). All 108 included patients had complete outcome data and were included in the final analysis (Figure 1). The baseline demographic and clinical characteristics were comparable between the two groups (Table 4). The mean age was 45.2 ± 12.6 years in Group A and 46.8 ± 11.9 years in Group B (p = 0.487). Male patients accounted for 59.3% and 55.6% in the treatment and control groups, respectively (p = 0.694). Baseline TCM syndrome scores, MCTSI scores, and laboratory parameters showed no significant differences between groups (all p > 0.05).

**TABLE 4:** Baseline demographic and clinical characteristics of the study population.

Characteristic	Group A (n = 54)	Group B (n = 54)	*p-value*
Age (years), mean ± SD	45.2 ± 12.6	46.8 ± 11.9	0.487
Male, n (%)	32 (59.3)	30 (55.6)	0.694
Body mass index (kg/m^2^), mean ± SD	24.8 ± 3.2	25.1 ± 3.5	0.632
Duration of symptoms (hours), mean ± SD	18.5 ± 8.3	19.2 ± 7.9	0.621
Etiology, n (%)	​	​	0.812
Biliary	22 (40.7)	24 (44.4)	​
Hyperlipidemia	12 (22.2)	10 (18.5)	​
Alcohol	10 (18.5)	9 (16.7)	​
Idiopathic	10 (18.5)	11 (20.4)	​
Baseline TCM syndrome score, mean ± SD	18.6 ± 3.4	18.2 ± 3.7	0.558
Baseline MCTSI score, mean ± SD	4.2 ± 1.3	4.1 ± 1.4	0.702
Baseline CRP (mg/L), mean ± SD	85.3 ± 28.6	82.7 ± 30.1	0.643
Baseline IL-6 (pg/mL), mean ± SD	42.5 ± 15.3	40.8 ± 14.7	0.548
Baseline WBC (×10^9^/L), mean ± SD	12.8 ± 3.5	12.5 ± 3.8	0.667
Baseline amylase (U/L), mean ± SD	385.2 ± 128.6	372.5 ± 135.4	0.612

SD: standard deviation; TCM: traditional chinese medicine; MCTSI: Modified CT, severity index; CRP: C-reactive protein; IL-6: Interleukin-6; WBC: white blood cell.

## Primary outcomes

### TCM syndrome scores

The TCM syndrome scores improved significantly in both groups over time. However, Group A demonstrated significantly greater improvements compared to Group B at day 3 (MD = −4.82, 95%CI: −6.15 to −3.49, p < 0.001) and before discharge (MD = −6.35, 95%CI: −7.89 to −4.81, p < 0.001) (Table 1). The between-group differences were clinically meaningful, indicating superior symptom relief with the integrated TCM approach.

### MCTSI scores

MCTSI scores decreased in both groups during hospitalization. The Group A showed significantly lower MCTSI scores at day 3 (MD = −1.25, 95%CI: −1.78 to −0.72, p < 0.001) and before discharge (MD = −1.68, 95%CI: −2.31 to −1.05, p < 0.001) compared to Group B (Table 2), suggesting faster resolution of pancreatic inflammation and reduced severity.

### Intestinal barrier function

Intestinal barrier function markers improved significantly in Group A compared to the control group. At day 3, plasma D-lactate (MD = −2.15, 95%CI: −2.89 to −1.41, p < 0.001), diamine oxidase (MD = −3.42, 95%CI: −4.28 to −2.56, p < 0.001), and endotoxin (MD = −0.18, 95%CI: −0.25 to −0.11, p < 0.001) levels were significantly lower in Group A (Table 5). These improvements were maintained before discharge, indicating sustained protective effects on intestinal barrier integrity.

**TABLE 5:** Comparison of intestinal barrier function markers between groups.

Marker	Time point	Group A (n = 54)	Group B (n = 54)	Mean difference (95%CI)	*p-value*
D-lactate (mg/L)	Baseline	8.5 ± 2.3	8.3 ± 2.5	0.2 (−0.7–1.1)	0.658
	Day 3	4.2 ± 1.5	6.4 ± 1.8	−2.15 (−2.89 to −1.41)	<0.001
	Before discharge	2.1 ± 0.9	4.5 ± 1.2	−2.35 (−2.89 to −1.81)	<0.001
Diamine oxidase (U/L)	Baseline	15.8 ± 4.2	16.1 ± 4.5	−0.3 (−1.9 to 1.3)	0.712
	Day 3	8.5 ± 2.8	11.9 ± 3.2	−3.42 (−4.28 to −2.56)	<0.001
	Before discharge	5.2 ± 1.6	9.8 ± 2.4	−4.62 (−5.36 to −3.88)	<0.001
Endotoxin (EU/mL)	Baseline	0.45 ± 0.12	0.43 ± 0.11	0.02 (−0.03–0.07)	0.428
	Day 3	0.22 ± 0.08	0.40 ± 0.10	−0.18 (−0.25 to −0.11)	<0.001
	Before discharge	0.12 ± 0.05	0.28 ± 0.09	−0.16 (−0.21 to −0.11)	<0.001

Data are presented as mean ± standard deviation. CI: confidence interval.

## Secondary outcomes

### Inflammatory markers

Significant reductions in inflammatory markers were observed in Group A compared to the control group. CRP levels at day 3 (MD = −28.5, 95%CI: −36.2 to −20.8, p < 0.001) and before discharge (MD = −35.2, 95%CI: −44.6 to −25.8, p < 0.001) were substantially lower in Group A (Table 6). Similarly, IL-6 levels showed significant reductions at day 3 (MD = −15.3, 95%CI: −19.7 to −10.9, p < 0.001) and before discharge (MD = −18.6, 95%CI: −23.5 to −13.7, p < 0.001). White blood cell counts and neutrophil percentages also improved significantly in the treatment group.

**TABLE 6:** Comparison of inflammatory markers between groups.

Marker	Time point	Group A (n = 54)	Group B (n = 54)	Mean difference (95%CI)	*p-value*
CRP (mg/L)	Baseline	85.3 ± 28.6	82.7 ± 30.1	2.6 (−8.9–14.1)	0.643
​	Day 3	28.5 ± 12.3	57.0 ± 18.5	−28.5 (−36.2 to −20.8)	<0.001
​	Before discharge	8.2 ± 4.5	43.4 ± 15.2	−35.2 (−44.6 to −25.8)	<0.001
IL-6 (pg/mL)	Baseline	42.5 ± 15.3	40.8 ± 14.7	1.7 (−4.9–8.3)	0.548
​	Day 3	15.2 ± 6.8	30.5 ± 10.2	−15.3 (−19.7 to −10.9)	<0.001
​	Before discharge	6.8 ± 3.2	25.4 ± 8.6	−18.6 (−23.5 to −13.7)	<0.001
WBC (×10^9^/L)	Baseline	12.8 ± 3.5	12.5 ± 3.8	0.3 (−1.2–1.8)	0.667
​	Day 3	7.5 ± 2.1	10.2 ± 2.8	−2.7 (−3.7 to −1.7)	<0.001
​	Before discharge	5.8 ± 1.5	8.5 ± 2.2	−2.7 (−3.5 to −1.9)	<0.001
Neutrophils (%)	Baseline	82.5 ± 6.8	81.8 ± 7.2	0.7 (−2.1–3.5)	0.623
​	Day 3	68.5 ± 5.2	78.2 ± 6.5	−9.7 (−12.1 to −7.3)	<0.001
​	Before discharge	58.2 ± 4.8	70.5 ± 5.8	−12.3 (−14.6 to −10.0)	<0.001

CRP: C-reactive protein; IL-6: Interleukin-6; WBC: White blood cell. Data are presented as mean ± standard deviation. CI: confidence interval.

### SIRS scores

**FIGURE 2:**
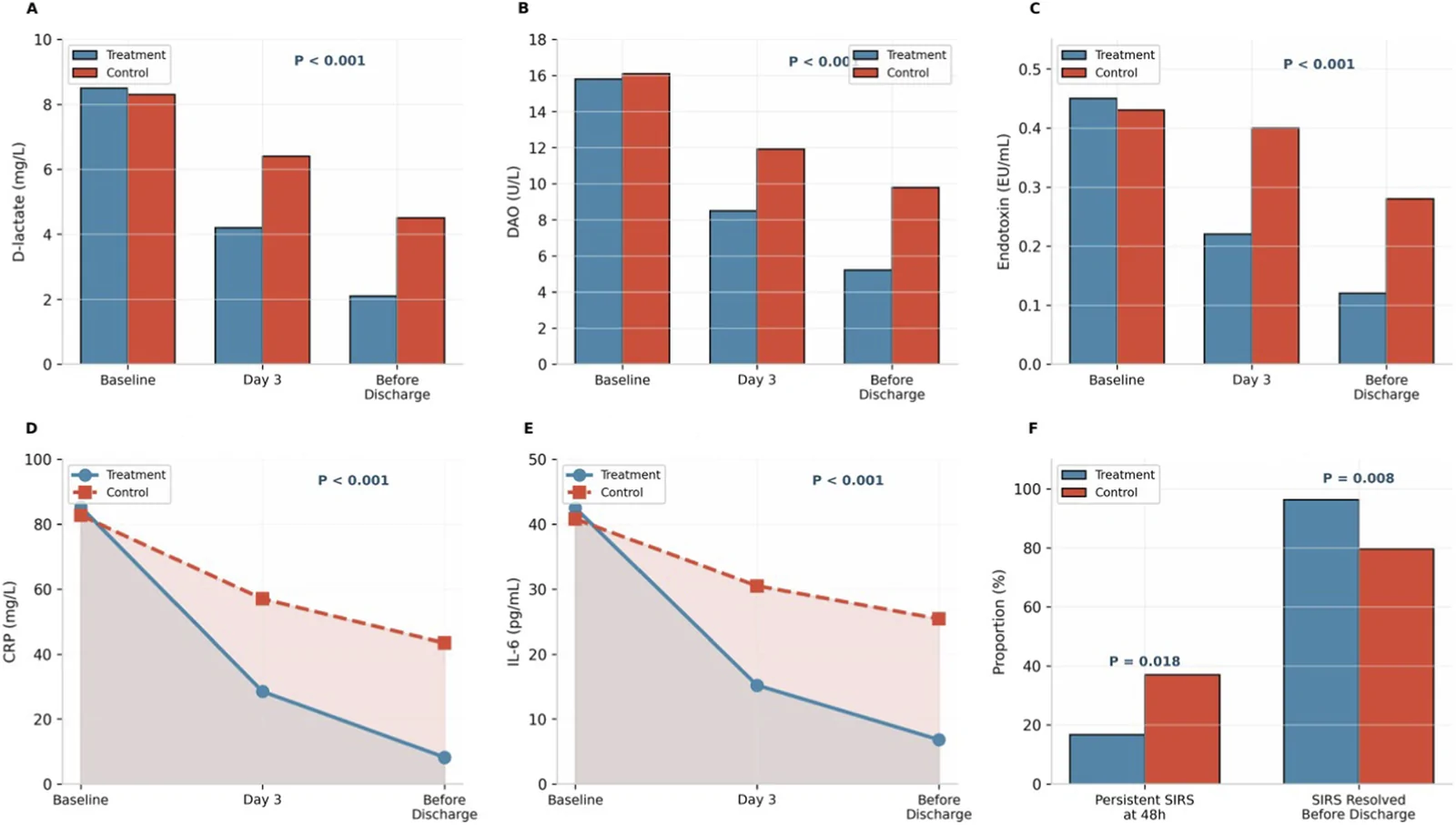
Intestinal barrier function and inflammatory response: mechanisms of sepsis prevention by Qingyi Decoction. **(A–C)** Intestinal barrier markers (D-lactate, DAO, endotoxin) showed significant improvement in Group A at day 3 and before discharge (p < 0.001), demonstrating effective gut barrier protection against bacterial translocation. **(D,E)** Inflammatory markers (CRP, IL-6) exhibited rapid and sustained reduction (p < 0.001). **(F)** SIRS resolution was significantly faster in Group A (p = 0.018 and p = 0.008). Error bars represent standard deviation.

Regarding additional secondary laboratory parameters, serum amylase levels normalized significantly faster in Group A compared to Group B (MD = −128.5 U/L at day 3, 95%CI: −165.2 to −91.8, p < 0.001). Serum creatinine and blood urea nitrogen showed improvement in both groups over the hospitalization period, without statistically significant between-group differences (creatinine: MD = −4.2 μmol/L, p = 0.312; BUN: MD = −0.8 mmol/L, p = 0.284 before discharge), consistent with the mild-to-moderate severity of the enrolled population and the absence of significant acute kidney injury in either group. SIRS scores improved significantly in both groups, with Group A showing faster resolution of systemic inflammatory response. At 48 h, the proportion of patients with persistent SIRS was significantly lower in Group A (16.7% vs. 37.0%, p = 0.018). Before discharge, 96.3% of patients in Group A had resolved SIRS compared to 79.6% in Group B (p = 0.008) (Table 3). Changes in intestinal barrier function, inflammatory markers, and SIRS resolution are summarized in Figure 2.

### Sepsis incidence

The incidence of sepsis was significantly lower in Group A compared to Group B (3.7% vs. 14.8%, OR = 0.22, 95%CI: 0.05–0.98, p = 0.046). This represents a 75% relative risk reduction in sepsis development with the integrated TCM therapy. The protective effect against sepsis was likely mediated through the combined mechanisms of improved intestinal barrier function (reduced D-lactate, DAO, and endotoxin), attenuated systemic inflammation (reduced CRP and IL-6), and faster SIRS resolution. Notably, Group A also showed lower MODS incidence (1.9% vs. 7.4%), ICU admission rate (5.6% vs. 16.7%), and mortality (0% vs. 3.7%), although these differences did not reach statistical significance, likely due to the relatively small sample size and low event rates in this mild-to-moderate AP population.

### Clinical outcomes

**FIGURE 3:**
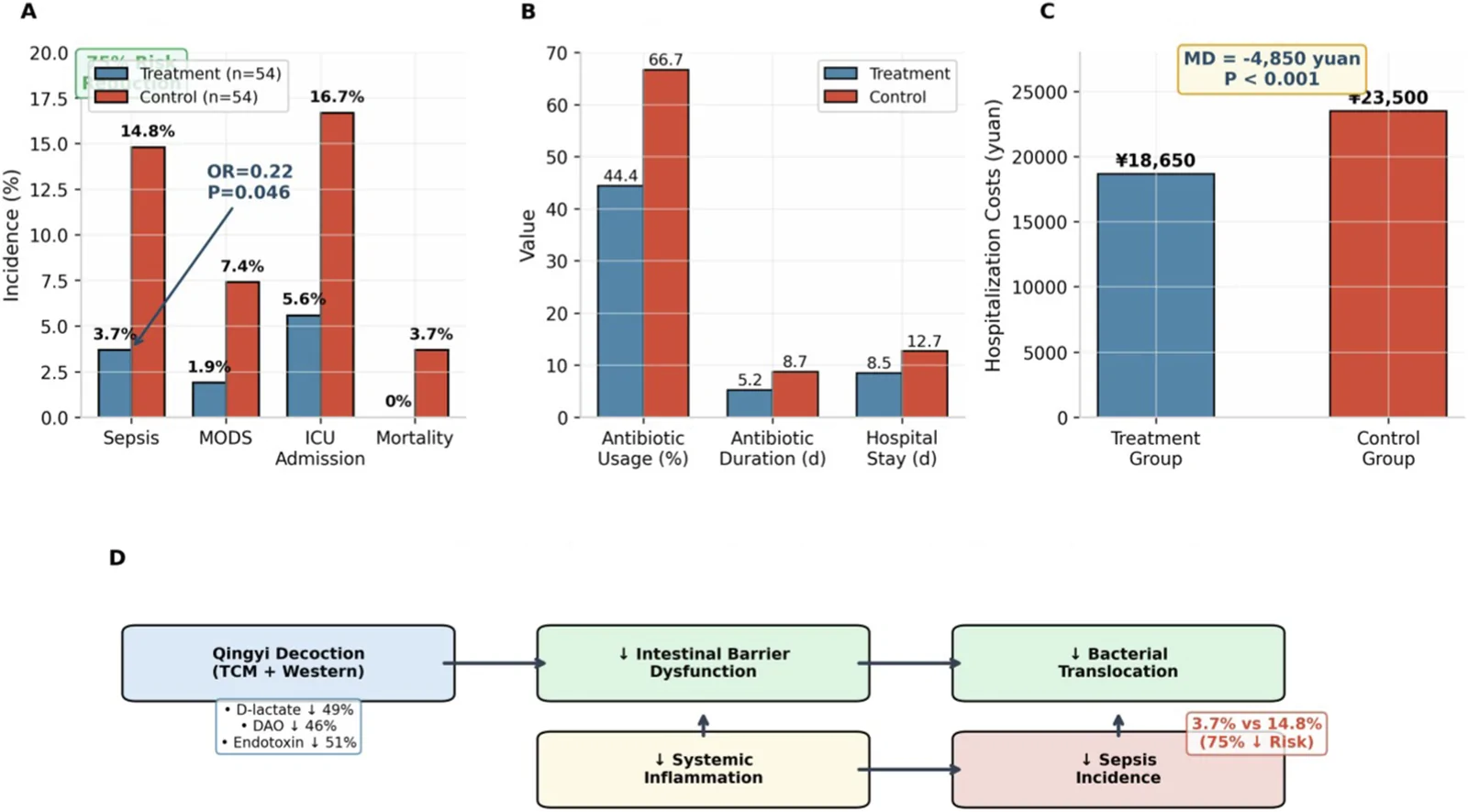
Sepsis prevention and clinical outcomes: efficacy of Qingyi Decoction in reducing sepsis burden. **(A)** Sepsis incidence was significantly lower in Group A (3.7% vs. 14.8%, OR = 0.22, p = 0.046), with a 75% relative risk reduction. MODS, ICU admission, and mortality rates were also lower. **(B)** Antibiotic usage, duration, and hospital stay were significantly improved. **(C)** Hospitalization costs were reduced by 4,850 yuan per patient (p < 0.001). **(D)** Proposed mechanism: Qingyi Decoction protects intestinal barrier function, reduces bacterial translocation and systemic inflammation, thereby preventing sepsis progression.

The Group A demonstrated significantly better clinical outcomes compared to Group B (Table 7). Antibiotic usage rate was significantly lower (44.4% vs. 66.7%, p = 0.021), and antibiotic duration was shorter (MD = -3.5 days, 95%CI: −4.8 to -2.2, p < 0.001). Length of hospital stay was significantly reduced (MD = -4.2 days, 95%CI: −5.6 to -2.8, p < 0.001), and hospitalization costs were lower (MD = -4,850 yuan [approximately USD 683], 95%CI: −6,320 to −3,380, p < 0.001). These findings suggest that sepsis prevention through intestinal barrier protection not only improves clinical safety but also yields substantial healthcare economic benefits. Sepsis incidence and related clinical outcomes are summarized in Figure 3.

**TABLE 7:** Comparison of sepsis incidence and clinical outcomes between groups.

Outcome	Group A (n = 54)	Group B (n = 54)	OR/MD (95%CI)	*p-value*
Sepsis incidence, n (%)	2 (3.7)	8 (14.8)	0.22 (0.05–0.98)	0.046
MODS incidence, n (%)	1 (1.9)	4 (7.4)	0.24 (0.03–2.12)	0.198
ICU admission, n (%)	3 (5.6)	9 (16.7)	0.29 (0.08–1.12)	0.072
Mortality, n (%)	0 (0)	2 (3.7)	-	0.496
Antibiotic usage rate, n (%)	24 (44.4)	36 (66.7)	0.41 (0.19–0.88)	0.021
Antibiotic duration (days), mean ± SD	5.2 ± 2.1	8.7 ± 3.5	−3.5 (−4.8 to −2.2)	<0.001
Length of hospital stay (days), mean ± SD	8.5 ± 2.8	12.7 ± 3.6	−4.2 (−5.6 to −2.8)	<0.001
Hospitalization costs (yuan; approximate USD equivalent at 7.1 RMB/USD, 2024–2025), mean ± SD	18,650 ± 3,850	23,500 ± 4,620	−4,850 (−6,320 to −3,380)	<0.001

OR: odds ratio; MD: mean difference; CI: confidence interval; MODS: multiple organ dysfunction syndrome; ICU: Intensive care unit. Data are presented as mean ± standard deviation or n (%).

### Safety analysis

No serious adverse events related to Qingyi Decoction were observed during the study. Two patients in Group A experienced mild diarrhea, which resolved after adjusting the Dahuang dosage. No significant differences were observed in liver and kidney function parameters between groups, indicating good safety profile of the integrated therapy.

## Discussion

This retrospective clinical study demonstrates that Qingyi Decoction combined with Western medicine comprehensive therapy significantly improves clinical outcomes in patients with mild to moderate acute pancreatitis. The integrated approach provided superior symptom relief, faster resolution of pancreatic inflammation, enhanced intestinal barrier function, reduced systemic inflammatory response, and most importantly, significantly decreased sepsis incidence compared to Western medicine alone.

The pathophysiology of acute pancreatitis involves a complex inflammatory cascade initiated by premature activation of pancreatic enzymes within the acinar cells, leading to autodigestion of the pancreas and surrounding tissues (Bhatia et al., 2005). This process is characterized by multiple cell death pathways, including apoptosis, necrosis, and pyroptosis, which release damage-associated molecular patterns (DAMPs) and pro-inflammatory cytokines, thereby amplifying the inflammatory response. The subsequent inflammatory response can trigger SIRS, which, if uncontrolled, progresses to sepsis and MODS. The gut plays a pivotal role in this process through bacterial translocation across the compromised intestinal barrier, serving as the “motor” of systemic inflammation and sepsis (Deitch, 1990). Intestinal epithelial cell death, particularly pyroptosis mediated by the NLRP3 inflammasome, contributes significantly to barrier disruption and bacterial translocation in severe AP. This “gut-lymph-pancreas” axis, coupled with dysregulated cell death pathways, represents a critical therapeutic target for sepsis prevention in AP.

Our findings demonstrate that Qingyi Decoction significantly improved intestinal barrier function, as evidenced by reduced levels of D-lactate, diamine oxidase, and endotoxin. D-lactate is a specific marker of intestinal permeability, as it is produced exclusively by bacterial fermentation in the intestinal lumen and enters the circulation only when the intestinal barrier is compromised (Sun et al., 2001). Diamine oxidase is an enzyme located in the intestinal mucosal epithelium, and its plasma levels reflect the integrity of the intestinal mucosal barrier (Peng et al., 2000). Endotoxin, a component of Gram-negative bacterial cell walls, is a potent trigger of systemic inflammation and its elevated plasma levels indicate bacterial translocation (Ammori, 2003). The improvement in these markers suggests that Qingyi Decoction may protect intestinal epithelial cells from excessive death, including apoptosis and pyroptosis, thereby maintaining barrier integrity. The significant reduction in these markers suggests that Qingyi Decoction effectively maintains intestinal barrier integrity, thereby preventing bacterial translocation and subsequent sepsis development. This mechanism is further supported by the significantly lower sepsis incidence in Group A (3.7% vs. 14.8%), representing a 75% relative risk reduction. The protective effect against sepsis is particularly clinically relevant, as sepsis is a major determinant of mortality in severe acute pancreatitis, with each episode of sepsis substantially increasing the risk of MODS and death (Frossard et al., 2008). It should be noted, however, that this finding is based on a small absolute number of events (2 vs. 8 cases) and the 95% confidence interval for the odds ratio (0.05–0.98) is wide with its lower bound approaching unity, indicating statistical fragility. This result should therefore be interpreted as a preliminary, exploratory finding rather than a definitive conclusion, and confirmation in larger prospective randomized controlled trials is warranted.

The anti-inflammatory effects of Qingyi Decoction were evidenced by significant reductions in CRP and IL-6 levels. CRP is an acute-phase reactant that reflects the severity of systemic inflammation, while IL-6 is a key pro-inflammatory cytokine that drives the inflammatory cascade in acute pancreatitis (Mayer et al., 2000). The rapid reduction of these markers in Group A suggests that the TCM formula effectively modulates the inflammatory response, potentially through multiple pathways including inhibition of nuclear factor-kappa B (NF-κB) activation and reduction of pro-inflammatory cytokine production (Rakonczay et al., 2008). Furthermore, emerging evidence suggests that Qingyi Decoction components, particularly emodin and baicalin, may modulate cell death pathways by inhibiting NLRP3 inflammasome activation and subsequent pyroptosis, as well as regulating mitochondrial-mediated apoptosis. The attenuation of systemic inflammation and modulation of cell death likely contribute to the lower SIRS persistence rate and reduced sepsis progression.

The composition of Qingyi Decoction follows the TCM principle of “treating blockage with passage” (tongze buyong) for the treatment of interior-heat-excess patterns. Dahuang (Rheum palmatum), the monarch herb used at a heavy dosage of 30g, functions to purge heat, break blood stasis, and promote bowel movement. Modern pharmacological studies have demonstrated that emodin, the active component of Dahuang, possesses anti-inflammatory, antioxidant, and intestinal protective properties (Werge et al., 2016). Importantly, emodin has been shown to inhibit NLRP3 inflammasome activation and subsequent pyroptosis in various cell types, including pancreatic acinar cells and intestinal epithelial cells. It also modulates mitochondrial function and reduces oxidative stress-induced apoptosis. Mangxiao (Natrii sulfas) enhances the purgative effect of Dahuang and softens hard stools. Zhishi (Citrus aurantium) and Houpo (Magnolia officinalis) promote qi movement and relieve abdominal distension.

Chaihu (Bupleurum chinense) and Huangqin (Scutellaria baicalensis) clear liver-gallbladder heat and regulate the Shaoyang channel. Baishao (Paeonia lactiflora) nourishes blood and relieves abdominal pain. Yinhua (*Lonicera japonica*) and Shengshigao (Gypsum fibrosum) clear heat and detoxify. Honghua (Carthamus tinctorius), Taoren (Prunus persica), and Chuanxiong (Ligusticum chuanxiong) activate blood circulation and remove blood stasis. Yanhusuo (Corydalis yanhusuo) and Chuanlianzi (Melia toosendan) relieve pain and regulate qi. Baicalin, the active flavonoid from Huangqin, has been demonstrated to exert protective effects against cell death by inhibiting oxidative stress-induced apoptosis and suppressing the NLRP3/caspase-1/GSDMD pyroptosis pathway. The combination of these herbs creates a synergistic effect that addresses multiple pathological aspects of acute pancreatitis, including the regulation of cell death pathways, collectively contributing to intestinal barrier protection and sepsis prevention.

The significant reduction in antibiotic usage rate and duration in Group A has important implications for antimicrobial stewardship. Overuse of antibiotics in acute pancreatitis has been associated with increased risk of fungal infections, antibiotic resistance, and higher treatment costs (Working Group IAP/APA Acute Pancreatitis Guidelines, 2013). By improving intestinal barrier function and reducing bacterial translocation, Qingyi Decoction may decrease the need for prophylactic or therapeutic antibiotics, thereby minimizing these risks. This antibiotic-sparing effect is directly linked to the prevention of sepsis, as reduced bacterial translocation lowers the likelihood of secondary infections that would otherwise necessitate broad-spectrum antibiotic therapy. The modulation of intestinal epithelial cell death by Qingyi Decoction components likely contributes to barrier restoration and reduced bacterial translocation.

The shortened hospital stay and reduced hospitalization costs observed in Group A have significant healthcare economic implications. With the increasing prevalence of acute pancreatitis and rising healthcare costs, cost-effective treatment strategies are essential. The integrated TCM approach not only improves clinical outcomes but also reduces the economic burden on patients and healthcare systems.

This study has several strengths. First, this retrospective design allowed for the evaluation of real-world clinical outcomes in a consecutive patient cohort. Second, the study employed comprehensive outcome measures, including TCM syndrome scores, imaging assessments, laboratory markers, and clinical outcomes, providing a holistic evaluation of treatment efficacy. Third, the study specifically focused on sepsis prevention as a primary clinical endpoint, with intestinal barrier function and cell death modulation as mechanistic targets, addressing a critical unmet need in AP management. Fourth, the study focused on a specific TCM syndrome pattern (intestinal damp-heat), ensuring homogeneity of the study population and enhancing the applicability of findings to clinical practice.

However, several limitations should be acknowledged. First, this was a single-center retrospective study, which may limit the generalizability of findings to other populations and healthcare settings. The retrospective design inherently carries risks of selection bias and confounding factors, although we attempted to minimize these through strict inclusion and exclusion criteria. Second, the study was open-label due to the nature of TCM interventions, which may introduce performance and detection bias. However, outcome assessors were blinded to treatment allocation to minimize bias. Third, the follow-up period was limited to hospitalization duration, and long-term outcomes such as recurrence rates, post-discharge sepsis, and quality of life were not assessed. Fourth, while we observed significant improvements in intestinal barrier function and clinical outcomes, direct measurement of cell death markers (such as caspase-3, caspase-1, GSDMD, and TUNEL staining) was not performed in this study. Future research should incorporate these molecular markers to confirm the modulation of cell death pathways by Qingyi Decoction. Fifth, the mechanism of action of Qingyi Decoction was not fully elucidated at the molecular level, and further studies are needed to investigate the specific signaling pathways involved in intestinal barrier protection and cell death modulation.

Future research should focus on several directions. First, prospective, multi-center, randomized controlled trials with larger sample sizes are needed to confirm the efficacy and safety of Qingyi Decoction in acute pancreatitis and sepsis prevention. Second, mechanistic studies using animal models and *in vitro* experiments should investigate the specific molecular targets and signaling pathways involved in the protective effects of Qingyi Decoction on intestinal barrier function, including tight junction proteins (occludin, claudin, ZO-1) and anti-inflammatory mechanisms (NF-κB, MAPK pathways). Furthermore, future studies should incorporate bioinformatics analysis and network pharmacology approaches to systematically identify the active components, potential targets, and molecular pathways through which Qingyi Decoction exerts its therapeutic effects. Network pharmacology methods, including target prediction (SwissTargetPrediction, TCMSP), protein-protein interaction (PPI) network construction, and KEGG/GO pathway enrichment analysis, can provide mechanistic hypotheses at the systems biology level. These computational findings can then be validated using molecular docking, *in vitro* cell experiments, and *in vivo* animal models, forming an integrated research paradigm combining computational prediction with experimental verification. Transcriptomic analysis (RNA-seq) of intestinal epithelial cells and pancreatic acinar cells before and after Qingyi Decoction treatment may reveal novel cell death-related gene signatures and regulatory networks. Third, detailed investigations into cell death modulation should be conducted, including assessment of apoptosis markers (caspase-3, Bax, Bcl-2), pyroptosis markers (NLRP3, caspase-1, GSDMD, IL-1β, IL-18), and necrosis markers (RIPK1, RIPK3, MLKL) in both pancreatic and intestinal tissues. Fourth, pharmacokinetic and pharmacodynamic studies should characterize the absorption, distribution, metabolism, and excretion of active components such as emodin and baicalin. Fifth, cost-effectiveness analyses should be conducted to evaluate the economic value of integrated TCM therapy in acute pancreatitis management and sepsis prevention.

## Conclusion

Qingyi Decoction combined with Western medicine comprehensive therapy significantly improves clinical symptoms, reduces inflammatory response, enhances intestinal barrier function, decreases sepsis incidence, and shortens hospitalization duration and costs in patients with mild to moderate acute pancreatitis. The integrated TCM approach represents a promising therapeutic strategy for AP management and sepsis prevention through intestinal barrier protection, with potential for widespread clinical application. These findings support the incorporation of TCM into standard treatment protocols for acute pancreatitis, particularly in regions where TCM is readily available and culturally accepted.

## Core tip

This retrospective clinical study demonstrates that Qingyi Decoction combined with Western medicine comprehensive therapy significantly improves clinical outcomes in patients with mild to moderate acute pancreatitis. The integrated approach provided superior symptom relief, faster resolution of pancreatic inflammation, enhanced intestinal barrier function, reduced systemic inflammatory response, modulation of cell death pathways, and most importantly, significantly decreased sepsis incidence compared to Western medicine alone.

## Data Availability

The raw data supporting the conclusions of this article will be made available by the authors, without undue reservation.
